# Small Non-Coding RNAs and Their Role in Locoregional Metastasis and Outcomes in Early-Stage Breast Cancer Patients

**DOI:** 10.3390/ijms25073982

**Published:** 2024-04-03

**Authors:** Daniel Escuin, Olga Bell, Bárbara García-Valdecasas, Montserrat Clos, Itziar Larrañaga, Laura López-Vilaró, Josefina Mora, Marta Andrés, Cristina Arqueros, Agustí Barnadas

**Affiliations:** 1 Institut de Recerca Sant Pau (IR Sant Pau), 08041 Barcelona, Spain; obell@santpau.cat (O.B.); bgvaldecasas@santpau.cat (B.G.-V.); mclos@santpau.cat (M.C.); ilarranaga@santpau.cat (I.L.); llopezv@santpau.cat (L.L.-V.); mandresg@santpau.cat (M.A.); carqueros@santpau.cat (C.A.); abarnadasm@santpau.cat (A.B.); 2 Hospital de la Santa Creu i Sant Pau, 08041 Barcelona, Spain; jmora@santpau.cat; 3 School of Medicine, Universitat Autònoma de Barcelona (UAB), 08193 Bellaterra, Spain; 4 Centro de Investigación Biomédica en Red Cáncer (CIBERONC), 28029 Madrid, Spain

**Keywords:** sncRNAs, snoRNAs, early breast cancer, sentinel lymph node, metastasis, biomarkers

## Abstract

Deregulation of small non-coding RNAs (sncRNAs) has been associated with the onset of metastasis. We evaluated the expression of sncRNAs in patients with early-stage breast cancer, performing RNA sequencing in 60 patients for whom tumor and sentinel lymph node (SLN) samples were available, and conducting differential expression, gene ontology, enrichment and survival analyses. Sequencing annotation classified most of the sncRNAs into small nucleolar RNA (snoRNAs, 70%) and small nuclear RNA (snRNA, 13%). Our results showed no significant differences in sncRNA expression between tumor or SLNs obtained from the same patient. Differential expression analysis showed down-regulation (n = 21) sncRNAs and up-regulation (n = 2) sncRNAs in patients with locoregional metastasis. The expression of *SNHG5*, *SNORD90*, *SCARNA2* and *SNORD78* differentiated luminal A from luminal B tumors, whereas *SNORD124* up-regulation was associated with luminal B HER2+ tumors. Discriminating analysis and receiver-operating curve analysis revealed a signature of six snoRNAs (*SNORD93*, *SNORA16A*, *SNORD113-6*, *SNORA7A*, *SNORA57* and *SNORA18A*) that distinguished patients with locoregional metastasis and predicted patient outcome. Gene ontology and Reactome pathway analysis showed an enrichment of biological processes associated with translation initiation, protein targeting to specific cell locations, and positive regulation of Wnt and NOTCH signaling pathways, commonly involved in the promotion of metastases. Our results point to the potential of several sncRNAs as surrogate markers of lymph node metastases and patient outcome in early-stage breast cancer patients. Further preclinical and clinical studies are required to understand the biological significance of the most significant sncRNAs and to validate our results in a larger cohort of patients.

## 1. Introduction

Cancer metastases are responsible for most breast cancer deaths. Despite intensive research in this field, our comprehension of the molecular events that drive metastatic progression remains largely incomplete. The identification of predictive and prognostic biomarkers is needed for early diagnosis of cancer and response monitoring of available therapies [1]. Ideally, these biomarkers should be highly specific and sensitive and several strategies are currently being developed to detect low expressed cancer-related biomarkers in liquid biopsies and other tumor samples [2]. However, more information on their expression in tumors, and thus suitability as biomarkers, is still required.

In recent years, small non-coding RNAs (sncRNAs) have emerged as important regulators of many cellular processes, including various steps of the metastatic process. SncRNAs encompass an abundant variety of RNA molecules, 15–300 nucleotides (nt) in length, including microRNAs (miRNAs), small interfering RNAs (siRNAs), piwi-interacting RNA (piRNAs), small nucleolar RNAs (snoRNAs) and small nuclear (snRNAs). 

SnoRNAs, which have long been known, represent a class of abundantly expressed sncRNAs, primarily present in the nucleolus and play pivotal roles in post-transcriptional rRNA processing and modification, thereby contributing significantly to the maintenance of cellular functions related to protein synthesis. SnoRNAs are approximately 30–300 nt long and, based on conserved sequence elements, are classified into C/D box snoRNAs (SNORDS) or H/ACA box snoRNA (SNORA), which determine the binding and modification of the RNA target. A third class of snoRNAs contains both sequence motifs and localizes to the nuclear Cajal bodies (SCARNA) [3]. However, approximately half of human snoRNA have no predictable rRNA targets, and numerous snoRNAs have been discovered to possess the ability to influence cell fate and alter disease progression; they therefore hold immense potential in terms of controlling human diseases, including Prader–Willi syndrome, Duplication15q syndrome and cancer [4]. It has been suggested that snoRNA dysregulation exhibits differential expression across various cancer types, stages, metastasis, treatment response and/or prognosis in patients [5]. This new role of snoRNAs has been addressed by recent studies showing that snoRNA can act to regulate pre-mRNA alternative splicing and mRNA abundance, as well as activate enzymes and be processed into shorter sncRNAs resembling miRNAs and piRNAs [6,7,8]. Furthermore, recent biochemical studies have shown that a given snoRNA can form both methylating and non-methylating ribonucleoprotein complexes, providing clues to the likely physical basis for such diverse new functions [9]. SnoRNAs are evidently more structurally and functionally diverse than previously thought, and their role in gene expression is under-appreciated. 

SnRNAs are a class of highly abundant sncRNA molecules with an average size of 150 nt present in the cell nucleus and are involved in intron removal from pre-mRNA. SnRNA form a large particulate complex (splicesome) along with ribonucleoprotein particles (snRNPs) and additional proteins, which binds to the primary RNA transcripts to mediate the splicing. Additional evidence indicates that snRNPs function in nuclear maturation of primary transcripts in mRNAs, gene expression regulation, splice donor in non-canonical systems and in 3′-end processing of replication-dependent histone mRNAs [10]. Accumulating evidence demonstrate that snRNA dysregulation are closely related to the progression of cancer through different mechanisms, such as transcriptional inhibition and post-transcriptional regulation [11].

In this study, we performed RNA sequencing to profile the sncRNA expression in 60 patients with early-stage breast cancer for whom tumor tissue and SLNs samples were available. We identified most of the sncRNAs as snoRNAs or snRNAs, and we found that, overall, down-regulation of snoRNAs was associated with patient locoregional metastatic status. Furthermore, our classifier model yielded a 6-snoRNA signature that clearly differentiated between negative and positive metastatic SLN and correlates with patient outcome. Deregulated snoRNAs showed a significant enrichment of biological processes associated with translation initiation, protein targeting to various organelles and regulation of Wnt and NOTCH signaling pathways. Our data highlight the potential use of sncRNAs as surrogate markers of locoregional metastases and patient outcome in breast cancer.

## 2. Results

### 2.1. Patients

For each of the 60 female patients who were included in this study, we analyzed paired tumor tissues and SLNs. The main clinicopathological characteristics of the patients are described in Table 1. Of the 60 patients, 40 (67%) had SLN-positive tumors, 20 were diagnosed as micrometastasis and 20 were diagnosed as macrometastasis.

### 2.2. RNA Sequencing

A total of 117 samples (98%) from 59 tumors and 58 SLNs passed the pre- and post-sequencing quality check, which confirmed average read quality and base quality Q-scores > 30 (99.9% correct) [12]. Three samples (1 tumor and 2 SLNs) were excluded from further analyses. In total, we analyzed 57 patients with paired samples (n = 117). The mean read number for tissues and SLNs were 3.8 million and 4.4 million, respectively. Following sequencing and trimming, reads were collapsed into a single read and passed into the analysis pipeline. This allowed for true quantification of the sncRNAs by eliminating library amplification bias and a better representation of the RNA molecules in the sample. We obtained an average 0.96 million and 1 million of collapsed reads for tissues and SLNs, respectively, and an average genome mapping rate of 25% and 23% for tissues and SLNs, respectively. The raw counts yield a total of 4207 sncRNAs that was reduced to 536 sncRNAs after performing a filtering step of at least 1 CPM in half of the samples. Count data were normalized and log2 transformed using the regularized log (rlog) method from the DESeq2 package (Table S1). The resulting sncRNAs were classified as snoRNAs (69.5%), snRNA (12.5%), miscellaneous RNAs (7.1%) and rRNAs (9.3%). Within the snoRNAs category, the majority of them were SNORDS (67.8%), followed by SNORA (29.7%) and SCARNA (2.6%).

### 2.3. Correlations and Clustering Analyses

**Figure 1:**
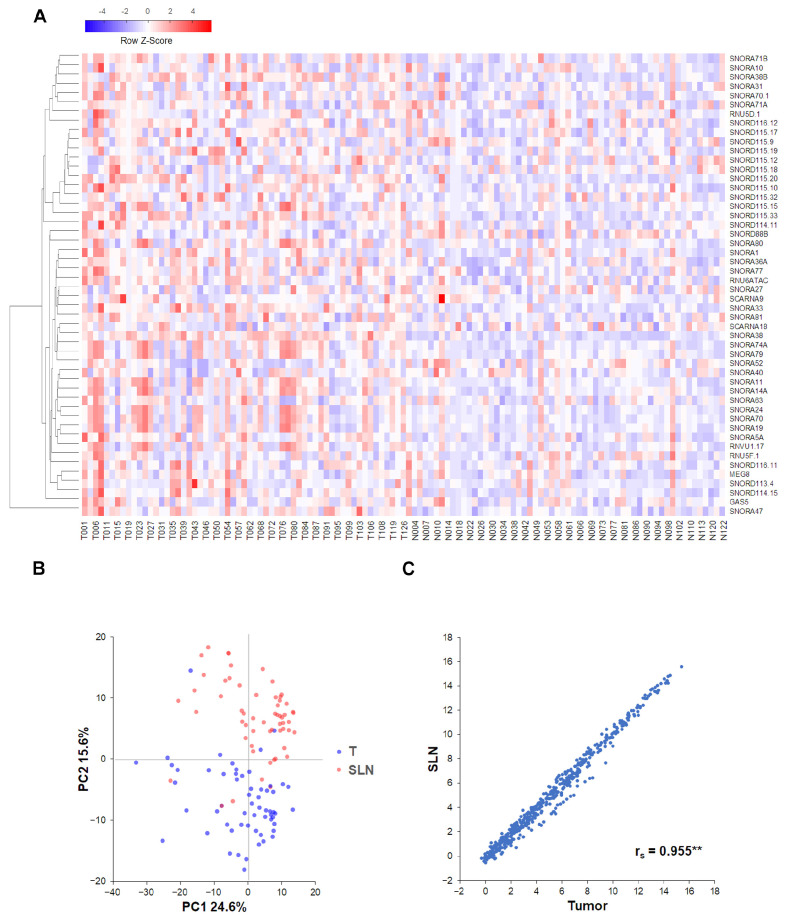
**Class discovery associated with SLN metastatic status.** The analysis was performed using 50 sncRNAs with the largest coefficient of variation based on rlog-normalized values. (**A**) Heatmap and unsupervised hierarchical clustering. Each row represents one sncRNA and each column represents one sample. The row Z-score scaling method was used to represent expression level above (red) and below (blue) the mean. (**B**) Principal component analysis shows sample clusters arising naturally based on the sncRNA expression profile. (**C**) Scatterplots depicting tumor-to-SLN comparison between samples from the same patient show the log expression of sncRNA expression for each sample type. The average Spearman’s correlation coefficient (r_s_) for all tumor–SLN comparisons is shown. ** Two-tailed *p* < 0.01.

To investigate whether patients were assigned into biological groups based on their sncRNA expression, we performed supervised hierarchical clustering using 50 sncRNAs with the largest coefficient of variation based on rlog-normalized counts. Our data indicated no significant differences between tumor and SLNs (Figure 1A). Similar results were obtained using a principal component analysis (PCA). Despite our data showing some differences between the two samples, those differences were not sufficiently large to cluster samples into different groups (Figure 1B). We also performed a tumor-to-SLN Spearman’s correlation analysis (r_s_). Our results showed that sncRNA expression in tumor and SLN samples from the same patient were highly correlated, with an average value for all patients of r_s_ = 0.955 (0.904–0.975) (Figure 1C, Table S2).

### 2.4. Differentially Expressed Tumor snoRNAs

**Figure 2:**
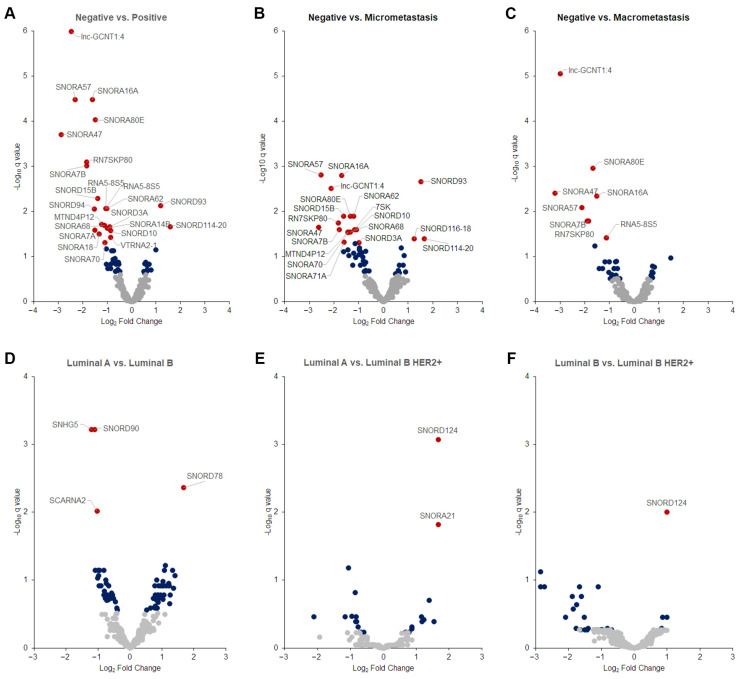
**Differentially expressed sncRNAs**. The volcano plots show differentially expressed sncRNAs in tumor samples according to patient locoregional metastatic status (**A**–**C**) and molecular subtype (**D**–**F**). The data show the logarithmic relationship between false discovery rate-adjusted *p* values (q value) (*y*-axis) and the log2 fold change expression (*x*-axis). Red, blue and grey dots show q values < 0.05, non-adjusted *p* values < 0.05 and non-significant *p* values > 0.05, respectively. Only snoRNAs with an absolute log2 fold change ≥ 1.5 are labeled.

Given the similarities in sncRNA expression between tumor and SLNs, we focused on tumor tissues to investigate whether differentially expressed (DE) sncRNAs were associated with the locoregional metastasis status of the patients. We first analyzed tumors samples according to their positive (n = 39) or negative (n = 20) metastasis status. We found 23 significantly DE sncRNAs (21 down-regulated and 2 up-regulated) after correcting for multiple testing (q < 0.05), with an absolute log2 fold change ≥ 1.5 associated with positive samples (Figure 2A) (Table S3). Similar results were obtained when patients were classified according to the SLN metastatic status, either positive macrometastasis (n = 19) or positive micrometastasis (n = 20) (Figure 2B,C). Interestingly, we observed that up-regulated sncRNAs were associated only with micrometastasis. No DE sncRNAs were found between patients with micrometastasis and macrometastasis. 

We next investigated DE sncRNAs based on breast cancer molecular subtypes. Our series included mainly patients with luminal A (n = 25) and luminal B (n = 21), followed by luminal B HER2+ (n = 8) and TN (n = 5) tumors (Table 1). Analyzing the first three subgroups (Figure 2D–F), our results show down-regulation of *SNHG5*, *SNORD90*, *SCARNA2* and up-regulation of *SNORD78* associated with luminal B compared to luminal A tumors. On the other hand, *SNORD124* up-regulation was associated with luminal B HER2+ compared to either luminal A (Figure 2E) or luminal B tumors (Figure 2F). 

### 2.5. Biological Significance and Enriched Analysis of sncRNAs

**Figure 3:**
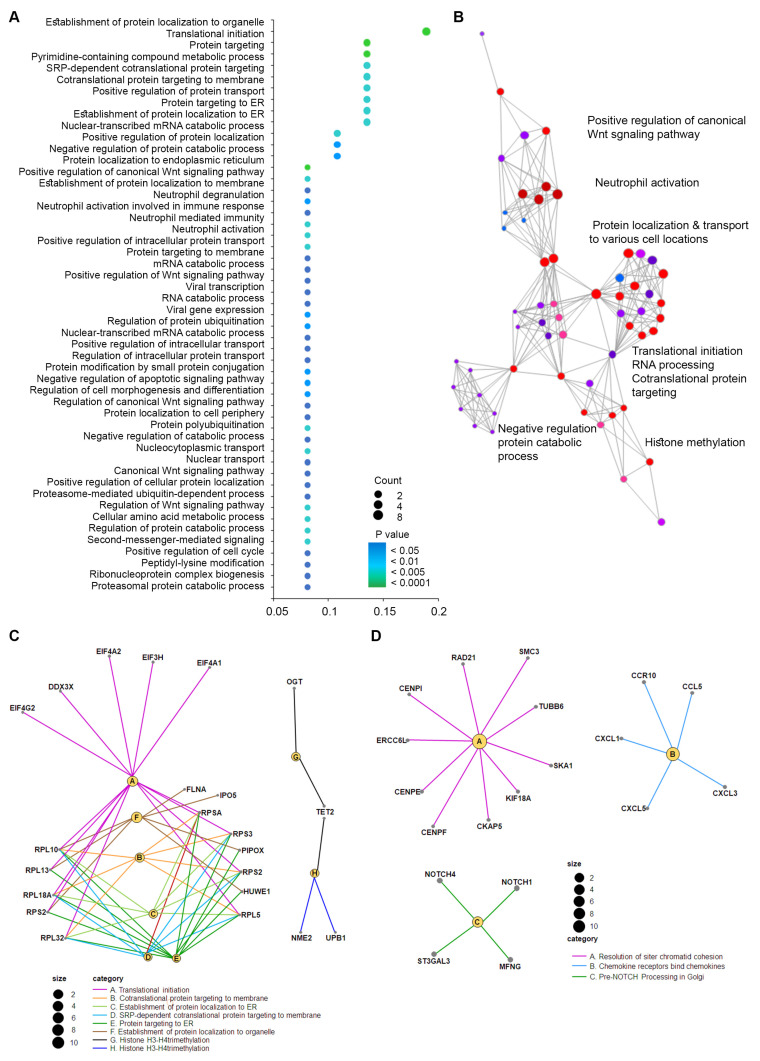
**Gene ontology (GO) enrichment analysis for significant biological processes associated with positive SLNs**. (**A**) The dot plot graph shows the 50 most significant biological process GO terms (*y*-axis) and the ratio between the number of expressed sncRNAs associated with the GO term and the number of significantly differentially expressed genes associated with the GO term (*x*-axis). The color of the nodes indicates the *p* value and the size of the nodes the number of sncRNAs associated with a specific GO term. (**B**) Enrichment map of the top 60 sncRNAs, with pathways grouped by similarity. Node size indicates the number of sncRNAs found in a pathway and node color reflects the significance of the *p* value. (**C**,**D**) The neural plots show the link between genes and terms associated with the most significant GO terms or Reactome pathways, respectively.

We performed a biological significance analysis using DE sncRNAs based on patient locoregional metastatic status (*p* < 0.05 and absolute log fold change > 0.3). In contrast to other sncRNAs such as microRNAs, annotation of snoRNAs in functional databases such as gene ontology (GO), KEGG or Reactome is scarce. Therefore, biological significance analysis was assessed using three different gene list, including the snoRNAs host genes and gene targets retrieved from the snoDB database, and genes correlated selected sncRNA expression in the TCGA-BRCA and SNOric databases (Table S4). Overall, our data show that the host genes of DE sncRNAs in patients with positive locoregional metastasis included GO categories associated with translational initiation, various processes targeting specific proteins to particular regions of the cell during or after the translational process, regulation of the Wnt signaling pathway (Figure 3A). Likewise, the top GO categories of targets genes associated with DE sncRNAs were also involved in the same processes. 

We carried out an enrichment analysis to determine those pathways associated with the DE sncRNAs. GO biological processes analysis using the SnoDB showed that the target genes were associated with Wnt signaling, protein translation, targeting proteins to particular cell locations, histone methylation, neutrophil activation and cell maturation and development (Figure 3B,C). To further understand the signaling pathways involved in the regulation of DE snoRNAs according to locoregional metastasis status, we performed a similar analysis using the Reactome, which, in contrast to the GO biological processes, makes extensive use of protein complex interactions in its representation, thus given a more detail picture of the pathways involved with a particular set of snoRNAs. Our results show an enrichment of DE sncRNAs associated with the NOTCH processing, resolution of sister chromatids and chemokine binding pathways (Figure 3D, Table 2).

### 2.6. Clinicopathological Correlation with DE sncRNAs

In our series of 60 patients with early-stage breast cancer, we observed recurrence in 11 (18%) patients. Median follow-up time was 9.6 years (range 0.4–12.5 years). At the last follow-up, nine (15%) patients were dead. The univariate analysis showed several sncRNAs associated with tumor grade (n = 134), lymphovascular invasion (n = 20) and tumor focality (n = 12) and, to a lesser degree, with menopausal status (n = 2) and tumor stage (n = 1) (Table 3 and Table S5).

**Figure 4:**
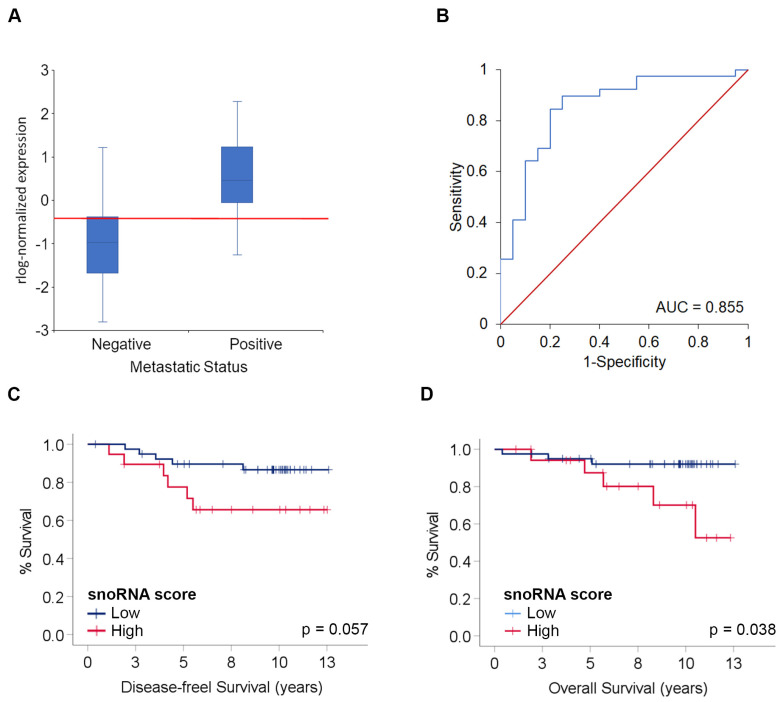
**Classifier model and association with patient outcome.** (**A**) Boxplot shows the normalized expression of the snoRNA score (*y*-axis) and the metastatic status of patients. The red line indicates the cutoff value. (**B**) ROC curve analysis of the snoRNAs score for discriminating patients with locoregional metastasis (blue). The reference line for random classification is shown in red. (**C**,**D**) Kaplan–Meier survival curves and log-rank tests for disease-free survival and overall survival based on snoRNAs categorized as low or high expression.

We further analyzed significant DE sncRNAs with an absolute log2 fold change ≥ 1.5 in terms of classifying patients according to the metastatic status. Various combinations of sncRNAs were examined using a classifier model producing a 6-snoRNA signature (*SNORD93*, *SNORA16A*, *SNORD113-6*, *SNORA7A*, *SNORA57* and *SNORA18A*) that clearly separated non-metastatic from metastatic tumors (Figure 4A). The receiver-operating characteristic (ROC) area under the curve (AUC) was 0.855, with a sensitivity of 89.7% and a specificity of 75% (Figure 4B). For results categorized into low or high expression based on the ROC analysis score, Kaplan–Meier analysis indicated that patients with low snoRNA score expression showed a trend toward improved disease-free survival (DFS), albeit not significant (HR = 3.012, 95% 0.92–9.92, *p* = 0.057) (Figure 4C) and better overall survival (HR = 4.067, 95% 1.06 0.92–9.92, *p* = 0.038) (Figure 4D).

## 3. Discussion

Breast cancer is one of the most prevalent cancers among women and the leading cause of cancer mortality in women [13]. Currently, LN affection remains the most important prognosis factor in breast cancer [14,15] and the presence of metastasis in the SLNs is still currently the recommended procedure for axillary staging of early breast cancer [16]. Our recent research has focused on the involvement of miRNAs in the development of locoregional metastases in patients with early-stage breast cancer [17,18,19]. We did not, however, investigate other classes of sncRNAs that have emerged in recent years as important regulators in cancer development and in the various steps of the metastatic process [20].

In this study, we investigated the expression of sncRNAs in paired primary tumor and SLNs from early-stage breast cancer patients and correlated the results with SLN metastatic status. Our RNA sequencing data show that 83% of the annotated sncRNAs were classified as snoRNAs (70%) or snRNAs (13%), whereas the rest belong to miscellaneous RNAs, including some long non-coding RNAs (lncRNAs). Nonetheless, the full landscape of sncRNAs may have not been revealed in our dataset as (1) we could only use tools as currently available for gene annotation are constantly improving (and those tools are constantly improving), (2) known RNA sequencing biases still exist [9] and (3) RNA library preparation limitations may prevent certain sncRNAs from being amplified [20].

Our data show that sncRNA expression in tumor and SLNs is similar, with minor non-significant changes that are likely due to histological differences between the SLN and the tumor, whereas SLNs are constituted mainly by lymphoid and monocytes cells the tumor tissue is formed mostly from epithelial and mesenchymal cells. Since our data showed high tumor–SLN correlation for patients, we performed further analyses on tumor samples.

We identified 23 DE sncRNAs associated with patient locoregional metastatic status. Overall, and similar to our previously reported data on miRNAs, we found that most DE sncRNAs were down-regulated (adjusted q < 0.05), suggesting that the expression of all sncRNAs follows a similar pattern in early-stage breast cancer patients [17,19]. Interestingly, down-regulated sncRNA expression was similar in patients affected by micro- and macrometastases, but up-regulated sncRNAs (*SNORD93*, *SNORD114-20* and *SNORD116-24*) was only significant in the micrometastatic group. However, the number of patients in each group was small (n = 20) and it remains to be elucidated whether loss of the expression of specific sncRNAs is part of the natural history course of breast cancer tumors.

Among the DE sncRNAs in our list, four snoRNAs (*SNORA47*, *SNORD94*, *SNORA70* and *SNORD10*) have been documented to be involved in various human cancers [5]. *SNORA47* has been reported to be up-regulated in human hepatocellular carcinoma and associated with intrahepatic metastasis and lymphatic invasion. In addition, a high expression of *SNORA47* predicted worse patient outcome [21]. *SNOR94*, *SNORA70* and *SNORD10* have been reported up-regulated in a p53 oncogenic gain-of-function mutant mouse osteosarcoma model. The authors showed by RNA-seq that a cluster of snoRNAs were highly up-regulated in p53 mutant tumors in association with the Ets2 transcription factor-binding site. Homozygous deletion of Est2 resulted in down-regulation of these snoRNAs and reversed the pro-metastatic phenotype of p53 mutant tumors [22]. The results for those four snoRNAs suggest that they act as oncogenes, contrast with our results and suggest that, in breast cancer *SNORA47*, *SNOR94*, *SNORA70* and *SNORD10* may play a role as a tumor-suppressor gene (TSG) by yet unknown mechanisms. In support of this argument, down-regulation of *SNORD10* has been associated with epigenetic promoter silencing in stage IV melanoma cell lines [23]. Thirteen sncRNAs in our dataset (some of which with a non-adjusted *p* < 0.05) (*RMRP*, *RN7SK*, *SNORA47*, *SNORA50C*, *SNORA71A*, *SNORA73B*, *SNORA7B*, *SNORA80E*, *SNORD10*, *SNORD112*, *SNORD12B*, *SNORD15A* and *VTRNA2-1*) were found annotated in the DisGeNet database [24], a curated database integrating information on human–disease associations from various repositories and inferred associations from literature text mining. *RMRP*, *SNORA7B*, *SNORD15A*, *SNORA71A* and *VTRNA2-1* have been previously reported in breast carcinomas [25,26,27,28]. *RMRP*, part of the RNase mitochondrial RNA processing (MRP) complex, has been found to be regulated by the oncogenic Wnt/b-catenin and Hippo/YAP pathways [25]. *SNORA7B* has been reported up-regulated in breast tumors compared to normal tissue [26], and *SNORD15A* and *SNORA71A* have been found up-regulated in brain metastases [27]. *VTRNA2-1* has been shown to be involved in the inhibition of protein kinase R (PKR) activity and act as a TSG in several cancer types. Increased breast cancer risk has been associated with down-regulation of *VTRNA2-1* linked to five methylation marks within the *VTRNA2-1* promoter region [28]. 

An interesting picture emerges from our data compared to the aforementioned reported data. First, our dataset included only tumor samples since normal tissue from the same patients was not available for study. We therefore could not address the relative expression of the DE sncRNAs found in our study compared to normal breast tissue, which would have help to elucidate whether the studied sncRNAs act as oncogenes or TSG. However, three DE sncRNAs associated with locoregional metastases *SNORA80E*, *SNOR15B* and *SNORD114-20* have been previously reported to be deregulated in invasive local BC compared to benign breast tissue [29]. Second, our biological significance and enrichment analyses—based on various databases that included host genes, target genes and interactions between genes and sncRNAs from the TCGA-BRCA atlas—showed similar signaling pathways as those described in the literature [20,30]. For instance, our data identified various GO biological processes as part of the Wnt signaling pathway. WNTs and WNT pathway components are also frequently over- or under-expressed in various cancers, and these changes are correlated with epigenetic regulation of promoter activity. In some contexts, both the canonical and non-canonical WNT signaling, which governs processes such as cell polarity and morphogenesis, may also contribute to tumor formation by promoting cell migration, invasiveness and metastasis [31]. In addition to the GO biological processes that focus on the activities of individual genes, we used the Reactome pathway, which makes extensive use of protein complexes in their pathway representations and describes their formation, dissociation and activities [32]. The main Reactome pathways were associated with the Notch signaling pathway (NSP), a highly conserved pathway for cell–cell communication involved in the regulation of cellular differentiation, proliferation, and specification [33]; chemokine receptors and their interaction with various chemokines that activates integrins for leukocyte adherence on endothelial cells and induces chemotaxis of leukocytes in tissue microenvironments [34]; and various processes associated with mitosis such as resolution of sister chromatids during mitotic prometaphase that indicates the involvement of sncRNAs in cell proliferation.

In addition to finding a correlation of the DE of sncRNAs with patient metastasis status, we found that a large number of sncRNAs correlated with various clinicopathological features, especially tumor grade, lymphovascular invasion, tumor focality and breast cancer molecular subtypes, in agreement with a recent study [29]. Interestingly, we have also described *SNORD124* up-regulation in tumors expressing HER2, suggesting that *SNORD124* could serve as a diagnostic biomarker for HER2-positive tumors. More importantly, our six-snoRNAs signature (*SNORD93*, *SNORA16A*, *SNORD113-6*, *SNORA7A*, *SNORA57* and *SNORA18A*) accurately distinguished between patients with locoregional metastasis. We also found that a low expression of the snoRNA discriminant score was associated with better patient outcome.

The main limitation of our study is the small number of samples used to assess the potential of sncRNAs as surrogate biomarkers of the lymph node metastasis in breast cancer. Therefore, our results are preliminary and must be interpreted with caution. Nonetheless, in this study, we provide evidence that several sncRNAs are associated with the locoregional metastatic status and patient outcome in early-stage breast cancer. Further studies are required in a larger number of patients to clinically validate our results and to unveil the molecular mechanisms of the sncRNAs described in this study.

## 4. Materials and Methods

**Patients**. We studied 60 patients with early-stage breast cancer treated with surgery. Male patients were excluded from this study. Sample size was determined according to the model developed by Dobin, K et al. [35]. None of the patients had been previously treated with surgery, chemotherapy or radiation. All patients had confirmed diagnoses based on tumor biopsy histopathology and intraoperative SLN tissue evaluated using the OSNA assay [36]. All tumors were invasive ductal carcinomas (IDCs) with or without an in situ component. The following clinical and pathological parameters were recorded: age, menopausal status, personal and family disease precedents, clinical follow-up, tumor stage determined according to the UICC system [37], histological grade determined using the Elston–Ellis grading system [38], tumor histology, presence of associated carcinoma in situ, presence of vascular and lymphatic invasion, tumor infiltrating lymphocytes, tumor focality, tumor necrosis; proliferation of non-tumoral tissue. For each patient, we collected paired tumor and SLNs (n = 120 samples). Samples were classified according to the SLN status as negative (n = 20) or positive (n = 40). Positive samples were sub-classified as macrometastatic (n = 20) or micrometastatic (n = 20) [36].

**RNA isolation.** Tumor and SLNs were processed as previously described [17]. RNA was isolated from tumor and SLNs samples using miRNeasy (Qiagen , Germantown, MD, USA) according to the manufacturer’s instructions an eluted in a volume of 30 μL. The RNA integrity (RIN) level was measured for each RNA sample using Agilent TapeStation (Santa Clara, CA, USA). All samples used in this study had a RIN value > 7. A range of spike-ins were added to all samples prior to RNA isolation. A Pre-sequencing quality check by q-PCR was performed on all samples to control for the quality of the RNA and inhibition in downstream enzymatic reactions as previously described [18].

**RNA sequencing**. All steps required to performed next-generation sequencing and genome annotation were performed as previously described [17,18]. Genome annotation was performed using the QIAGEN CLC Genomics Server v20.0.4 (Qiagen, Germantown, MD, USA). Following sequencing, Cutadapt (1.9.1) [39] was used to trim adaptor sequences. A quality check was performed to ensure Q-scores > 30 (>99.9% correct) for our data [12]. Reads with the correct length were collapsed into FASTQ files. Bowtie2 software (2.2.6) was used to map the reads. The mapping criterion for aligning reads to spike-ins, abundant sequences and databases was for reads to perfectly match the reference sequences. To map the genome, two mismatches were allowed in the sequences. Small insertions and deletions were not allowed. The resulting sequences were annotated using the human assembly GRCh38 (Ensembl) and the snoDB database v1.2.1 [40]. The raw data were filtered to keep only sncRNAs with at least 1 CPM in half of the samples. Count data were then normalized and log2 transformed using the regularized log (rlog) method from the DESeq2 package [41] to eliminate biases in the composition of the sequencing libraries and to stabilize variance–mean dependence in count data.

**Correlation, hierarchical clustering and differential expression analyses.** Correlation analyses were performed using the rlog-normalized counts matrix. Spearman’s rho (r_s_) statistic and a heatmap plotted with Euclidian distances were used to measure similarities between samples from the same patients. To visualize sample expression profiles, hierarchical clustering was performed using Euclidian distances and scaled and centered rlog-normalized counts. PCA was performed to reduce the rlog-normalized counts in two dimensions. Differential expression analyses were performed using the trimmed mean of M values (TMM) normalization method [42], converted to log2 scale, the R statistical software package v3.6.3 and libraries from the Bioconductor Project (www.bioconductor.org, accessed on 26 March 2024) [43].

**Biological significance and enrichment analyses**. Functional annotation of selected sncRNAs with a *p* < 0.05 was performed using Ensembl, NCBI resources and snoDB database v1.2.1 [40]. Validated gene targets were searched for using R software v3.6.3 to retrieve sncRNA-target interactions from DisGeNet v7.0 database [24]. The biological significance analysis was performed using gene and host set lists from the snoDB database and the TCGA_BRCA dataset from the SNORic [44] data portal. Enrichment analyses was conducted using GO [45] and the Reactome pathway database [46]. The analyses were performed using the R/Bioconductor’s cluster Profiler package v3.12.0 [47].

**Classifier model building.** Briefly, several iterations were performed during the resampling step in a balanced, random manner. The data were split into training and test cohorts, which were used to build and validate the model, respectively. Within each iteration, biomarker candidates were selected using different methods (*t*-test, lasso, random forest), and choosing a fixed number of features (3, 5, 10 and 25). Once candidates were selected, classification profiles were created using penalized logistic regression, partial least squares-linear discriminant analysis, and support vector machines to find the best candidate in terms of accuracy and robustness of the data. A ROC analysis was performed to assess diagnostic score sensitivity and specificity. Optimization of the linear combination of biomarkers, was performed using the total area under the ROC curve (AUC) [48,49].

**Statistics**. Differentially expressed snoRNAs from RNA sequencing data were detected by an exact test based on conditional maximum likelihood included in the R Bioconductor edgeR package [50]. *p* values from RNA sequencing were corrected (q values) for multiple testing using the Benjamini–Hochberg procedure [51]. A false discovery rate q < 0.05 was considered significant. In all group comparisons, missing expression values were treated as zero. Differences in total snoRNAs numbers between groups were analyzed by using two-sided parametric *t*-tests. Clinicopathological analysis was performed using Student’s T-test to compare quantitative variables and the X2 or Fisher exact tests to compare qualitative variables. DFS was defined as the time from diagnosis to either date of first relapse (local, regional, contralateral or metastatic) or second primary cancer. OS was defined as the time from sample collection to death from any cause. Patients lost to follow-up were censored at the last contact. Kaplan–Meier and log-rank analyses were used to compare DFS and OS and a Cox regression model was used to perform the multivariate analysis. A two-sided *p* value ≤ 0.05 was considered significant.

## Figures and Tables

**Table 1:** Basic patient and tumor characteristics.

Variable		Total (%)	LN Negative (%)	LN Positive (%)
Patients (%)		60 (100)	20 (100)	40 (100)
Age (years)	Mean + SD	63.6 ± 14.2	68.8 ± 11.9	60.9 ± 14.7
	Median (range)	64.3 (26–88)	71 (47–87)	60 (26–88)
	<40 years	3 (5)	0 (0)	3 (8)
	40–50 years	6 10)	2 (10)	4 (10)
	>50 years	51 (85)	18 (90)	33 (82)
Tumor status	T1c	32 (53)	12 (60)	20 (50)
	T2	28 (47)	8 (40)	19 (50)
Node status	N0	20 (33)	20 (100)	0 (0)
	N1	40 (67)	0 (0)	40 (100)
Tumor stage	IA	12 (20)	12 (60)	0 (0)
	IIA	28 (47)	8 (40)	20 (50)
	IIB	20 (33)	0 (0)	20 (50)
Tumor grade	1	3 (5)	1 (5)	2 (5)
	2	34 (57)	14 (70)	20 (50)
	3	23 (38)	5 (25)	18 (45)
Tumor focality	Unifocal	38 (63)	13 (65)	25 (62)
	Multifocal	19 (32)	7 (35)	12 (30)
	Multicentric	3 (5)	0 (0)	3 (8)
Ki67 status	≤14%	29 (48)	7 (35)	22 (55)
	>14%	31 (52)	13 (65)	58 (18)
ER status	Negative	6 (10)	2 (10)	4 (10)
	Positive	54 (90)	18 (90)	36 (90)
PR status	Negative	22 (37)	6 (30)	16 (40)
	Positive	38 (63)	14 (70)	24 (60)
Molecular subtype	Luminal A	25 (42)	6 (30)	19 (48)
	Luminal B	21 (35)	10 (50)	11 (28)
	Luminal B HER2+	8 (13)	3 (15)	6 (15)
	HER2+	0 (0)	0 (0)	0 (0)
	TN	5 (8)	1 (5)	4 (9)
LVI	Negative	52 (87)	19 (95)	33 (83)
	Positive	8 (13)	1 (5)	7 (17)
Menopausal status	Premenopausal	10 (17)	2 (10)	8 (20)
	Postmenopausal	50 (83)	18 (90)	32 (80)
Breast affected	Left	28 (47)	10 (50)	18 (45)
	Right	32 (53)	10 (50)	22 (55)
Breast surgery	Mastectomy	18 (32)	5 (25)	13 (33)
	Lumpectomy	41 (68)	15 (75)	27 (67)

LN: lymph node; ER: estrogen receptor; PR: progesterone receptor; TN: triple negative; LVI: lymphovascular invasion.

**Table 2:** GO and Reactome enrichment analyses associated with Wnt and NOTCH signaling pathways. Data show sncRNAs associated with each biological term and their target genes.

ID	Description	Ratio	*p*	sncRNAs	Target Gene
GO:0090263	Positive regulation of canonical Wnt signaling pathway	3/37	0.003	*SNORA80E, SNORD10, SNORD15A*	*PSMD11, LGR5, DDX3X*
GO:0030177	Positive regulation of Wnt signaling pathway	3/37	0.005	*SNORA80E, SNORD10, SNORD15A*	*PSMD11, LGR5, DDX3X*
GO:0060070	Canonical Wnt signaling pathway	3/37	0.026	*SNORA80E, SNORD10, SNORD15A*	*PSMD11, LGR5, DDX3X*
GO:0016055	Wnt signaling pathway	3/37	0.083	*SNORA80E, SNORD10, SNORD15A*	*PSMD11, LGR5, DDX3X*
GO:0198738	Cell–cell signaling by wnt	3/37	0.084	*SNORA80E, SNORD10, SNORD15A*	*PSMD11, LGR5, DDX3X*
GO:0007219	Notch signaling pathway	12/474	0.003	*SNORD15B, SNORA68, SNORD10, SNORD93*	*DLL1, EGFL7, MFNG, MIB2, NOTCH4, NOTCH1, DNER, FOXA1, FOXC1, YBX1, TBX2, DLGAP5*
R-HSA-8951430	RUNX3 regulates WNT signaling	2/305	0.021	*SNORD93*	*RUNX3, TCF7L1*
R-HSA-157118	Signaling by NOTCH	14/305	0.008	*SNORD15B, SNORA68, SNORD10, SNORD93*	*DLL1, HDAC7, MFNG, MIB2, NOTCH4, PSMD12, YWHAZ, DNER, NOTCH1, TLE4, YBX1, FLT4, DLGAP5, ST3GAL3*
R-HSA-9012852	Signaling by NOTCH3	5/305	0.013	*SNORD15B, SNORA68, SNORD10, SNORD93*	*DLL1, NOTCH1, YBX1, DLGAP5, MIB2*
R-HSA-2691230	Signaling by NOTCH1 HD Domain Mutants in Cancer	3/305	0.008	*SNORD15B, SNORA68, SNORD10, SNORD93*	*DLL1, NOTCH1, MIB2*
R-HSA-2691232	Constitutive Signaling by NOTCH1 HD Domain Mutants	3/305	0.008	*SNORD15B, SNORA68, SNORD10, SNORD93*	*DLL1, NOTCH1, MIB2*
R-HSA-9013695	NOTCH4 Intracellular Domain Regulates Transcription	3/305	0.019	*SNORD15B, SNORA68, SNORD10, SNORD93*	*NOTCH4, NOTCH1, FLT4*
R-HSA-9013507	NOTCH3 Activation and Transmission of Signal to the Nucleus	3/305	0.034	*SNORD15B, SNORA68, SNORD10, SNORD93*	*DLL1, YBX1, MIB2*
R-HSA-350054	Notch-HLH transcription pathway	3/305	0.045	*SNORD15B, SNORA68, SNORD10, SNORD93*	*HDAC7, NOTCH4, NOTCH1*
R-HSA-1912399	Pre-NOTCH Processing in the Endoplasmic Reticulum	2/305	0.011	*SNORD15B, SNORA68, SNORD10, SNORD93*	*NOTCH4, NOTCH1*
R-HSA-2660825	Signaling by NOTCH1 t(7;9) (M1580_K2555) Translocation Mutant	2/305	0.016	*SNORD15B, SNORA68, SNORD10/SNORD93*	*DLL1, NOTCH1*
R-HSA-2660826	Constitutive Signaling by NOTCH1 t(7;9)(M1580_K2555) Translocation Mutant	2/305	0.016	*SNORD15B, SNORA68, SNORD10/SNORD93*	*DLL1, NOTCH1*
R-HSA-9013700	NOTCH4 Activation of Signal to the Nucleus	2/305	0.038	*SNORD15B, SNORA68, SNORD10*	*NOTCH4, YWHAZ*

**Table 3:** Univariate analysis shows the number of significant (q < 0.01) sncRNAs associated with the patient clinicopathological characteristics of the patients. The names of the top 10 most significant sncRNAs are shown.

Variable	N	sncRNA Name
Tumor grade	134	*SNORD105B, SNORD19C, SNORD101, SNORD102, SNORD107, SNORD114-3, SNORD126, SNORD18A, SNORD61, SNORD64*
Lymphovascular invasion	20	*RNU5D-1, RNU5E-1, RNU7-1, SNORA15B-1, SNORA2B, SNORA36B, SNORA36C, SNORA38B, SNORA41, SNORA53*
Tumor focality	12	*RNU5D-1, RNU5E-1, RNU7-1, SNORA15B-1, SNORA2B, SNORA36B, SNORA36C, SNORA38B, SNORA41*
Menopausal status	2	*RNU5E-1, RNU5D-1*
Tumor stage	1	*SNORD115-8*

## Data Availability

The original contributions presented in this study are publicly available at the Sequence Research Archive under ID PRJNA663033 and are available for download here http://www.ncbi.nlm.nih.gov/bioproject/663033 (accessed on 26 March 2024).

## Supplementary Materials

The following supporting information can be downloaded at: https://www.mdpi.com/article/10.3390/ijms25073982/s1.
