# Supplementary material for: Small Non-Coding RNAs and Their Role in Locoregional Metastasis and Outcomes in Early-Stage Breast Cancer Patients
Source: Int J Mol Sci. 2024 Apr 3;25(7):3982. doi: 10.3390/ijms25073982 (PMC11011815; doi:10.3390/ijms25073982)
Supplement: Supplementary file 1 [file ijms-25-03982-s001.zip › Supplementary Tables Legends.pdf]

Supplementary Data

Table S1. Normalized expression of sncRNAs. Matrix of sncRNAs mapped and counted using the Ensembl GCRh38.p14 ncRNA database. Count data was normalized using the regularized log (rlog) method from the DESeq2 package and log2-transformed to correct for differences in sequencing depth and to quantified each RNA species.

Table S2. Correlation analysis. The Spearman's rho correlation was applied on tumor and SLN from the same patient using the rlog-normalized expression of sncRNAs. Data shows the correlation coefficient and the 2-tailed p value (highlighted in yellow) for each paired of samples.

Table S3. Differentially expressed ncRNAs. Data shows the most significant differentially expressed sncRNAs for each comparison according to the metastatic status of patients (sheets 1-3) and the tumor molecular subtype (sheets 4-6). Log2 fold change, non-adjusted p-values and corrected q-values are shown.

Table S4. Biological significance and enrichment analysis. The data shows a complete list of all the biological processes associated with the differentially expressed sncRNAs based on the locoregional metastatic status of breast cancer patients. Data includes a list of snoRNA host genes and target genes retrieved from snoDB and a list of genes that correlated with the expression of snoRNAs in the TCGA-BRCA dataset based on the SNOric database.

Table S5. Association of clinicopathological features with sncRNA expression. Data shows univariate analysis of significant sncRNAs (adjusted q values <0.05) with clinicopathological characteristics of patients.
